# Comparison of Fecal Microbiome of Wild Red Deer in Different Regions Reveals Geographic Differentiation

**DOI:** 10.3390/microorganisms14102404

**Published:** 2026-10-20

**Authors:** Sibo Chen, Songze Li, Yongxiang Li, Yunxi Zhang, Xing Duan, Chonghui Xu, Wenxi Qian, Huazhe Si, Hanlu Liu, Zhipeng Li

**Affiliations:** 1 College of Forestry and Grassland Science, Jilin Agricultural University, Changchun 130118, China; 2 College of Forestry and Prataculture, Ningxia University, Yinchuan 095000, China; 3 College of Animal Science and Technology, Jilin Agricultural University, Changchun 130118, China; 4 College of Animal Science and Technology, Tarim University, Alar 843300, China; 5 College of Agriculture, Chifeng University, Chifeng 024000, China; 6 Joint International Research Laboratory of Modern Agricultural Technology, Ministry of Education, Jilin Agricultural University, Changchun 130118, China; 7 Key Lab of Animal Production, Product Quality and Security, Ministry of Education, Jilin Agricultural University, Changchun 130118, China

**Keywords:** Cervidae, microbial community, metagenomics, CAZymes, geographic differentiation

## Abstract

The gut microbiome plays critical roles in nutrient metabolism and host adaptation in ruminants, yet genome-level functional differences in the fecal microbiomes across geographic populations of wild red deer remain underexplored. Here, we integrated 16S rRNA gene sequencing and shotgun metagenomics to characterize the fecal microbiome of wild red deer populations in Inner Mongolia (NMG, *n* = 16) and Gansu Province (GS, *n* = 16), China. The NMG population exhibited significantly higher α-diversity and a distinct microbial community compared to GS (adjusted *p* < 0.05). NMG was enriched in *Clostridium*, *Eubacterium*, and *Bacillus*, while GS harbored higher abundances of *Ruminococcus*, *Oscillospira*, and *Faecalibacterium* (adjusted *p* < 0.05). Functional profiling revealed that the fecal microbiome in NMG showed greater functional potential associated with amino acid, pyruvate, and butanoate metabolism pathways, whereas the fecal microbiome in GS was enriched in the genetic potential of starch/sucrose metabolism, glycan degradation, and bile acid biotransformation (adjusted *p* < 0.05). CAZyme analysis demonstrated that GS was enriched in glycoside hydrolases, polysaccharide lyases, and carbohydrate esterases, while NMG showed higher auxiliary activity family abundance (adjusted *p* < 0.05). Genome-resolved metagenomics recovered 456 species-level genome bins (SGBs), revealing that GS-enriched SGBs (Rikenellaceae, Oscillospirales CAG-272) contained CAZyme genes associated with pectin degradation, whereas NMG-enriched SGBs contained genes associated with lignin modification and chitin degradation. These findings demonstrate the significant differences in the fecal microbiomes between the two geographically distinct wild red deer populations.

## 1. Introduction

Ruminants have evolved a complex and highly specialized gastrointestinal ecosystem that enables them to efficiently extract energy from plant structural carbohydrates, including cellulose, hemicellulose, and pectin [1]. This remarkable digestive capacity is mediated by dense and diverse microbial communities residing in the gastrointestinal tract, which harbor genes encoding carbohydrate-active enzymes (CAZymes) and also supply the host with essential amino acids and vitamins [2,3]. Accumulating evidence has demonstrated that the gut microbiome also plays roles in host adaptation to environmental changes. For example, a comprehensive analysis revealed the coevolutionary relationship between the gut microbiome and cervids in the plateau with enhanced energy metabolism capabilities, highlighting the contribution of the gut microbiome to the plateau adaptability of cervids [4]. The convergent evolution of high relative abundances of CAZymes has been observed in the gut microbiome of plateau-dwelling ungulates [5], highlighting the importance of the gut microbiome in enabling hosts to thrive under extreme conditions.

Cervidae represents the second most diverse lineage of ruminants, comprising 46 species distributed across tropical rainforests, temperate forests, alpine meadows, and arid and semi-arid environments [6,7]. Among cervids, red deer (*Cervus elaphus*) is one of the most widely distributed species, inhabiting temperate and subarctic regions across Eurasia and North America [8,9]. In China, wild red deer occur primarily in two ecologically contrasting regions [10]. Red deer in Inner Mongolia typically inhabit temperate mixed coniferous–broadleaf forests and forest–steppe transition zones, where their diet is dominated by woody browse, shrub shoots, and fibrous grasses [11,12]. In contrast, red deer in northwestern China (e.g., Gansu Province) mainly occupy alpine meadows and montane forest edges, where they consume diets richer in herbaceous plants, sedges, and alpine forbs [13,14,15,16]. Previous studies have investigated the fecal microbiome of wild and farmed red deer in NMG and GS, revealing differences in microbial composition that are associated with plant fiber utilization and lipid metabolism [14,17]. Moreover, in wild ruminants, the gut microbiome is expected to be sensitive to variations in dietary resources and habitat conditions, as these animals rely on naturally available plant materials whose composition and nutritional quality vary across ecosystems [18,19,20]. However, the differences in the fecal microbiome of wild red deer between NMG and GS remain unclear. Comparison of the gut microbiome between NMG and GS likely provides insights into the potential adaptive microbial responses.

Previous studies of cervids, including red deer, reindeer (*Rangifer tarandus*), and white-tailed deer (*Odocoileus virginianus*), have demonstrated that the gut microbial community is associated with host species, habitat characteristics, and dietary sources using 16S rRNA gene sequencing [20,21,22,23,24], which provides limited insight into microbial functional potential. Advances in shotgun metagenomics and genome-resolved binning have revolutionized our understanding of gut microbial ecology by enabling the reconstruction of metagenome-assembled genomes (MAGs) [25]. Metagenomic analyses have revealed the response of secondary bile acid synthesis and lipid metabolism in the fecal microbiome of wild red deer during winter [19], and that the dopaminergic synapse pathway of the fecal microbiome might play important roles in the adaptation of wild red deer to high altitudes [14]. These results highlighted the importance of examining the differences in gut microbial functional potential between NMG and GS red deer. Fecal sampling, as a non-invasive approach that minimizes disturbance to wildlife, has been widely adopted to characterize the gut microbiome of free-ranging animals, and the fecal microbiome has been shown to reflect, to a considerable extent, the functional characteristics of the hindgut microbiome [26,27]. However, the fecal microbiome does not fully represent the whole gastrointestinal tract microbiome or the rumen microbiome [25,28]. Despite these advances, it remains unclear whether and how the fecal microbial functional potentials vary between NMG and GS red deer.

Here, we integrated 16S rRNA gene amplicon sequencing, shotgun metagenomic sequencing, and genome-resolved binning to characterize the fecal microbiome of wild red deer from Inner Mongolia (NMG) and Gansu Province (GS), China, and we aimed to (1) compare the taxonomic composition and alpha diversity of fecal microbial communities between the two populations; (2) profile the abundance and diversity of CAZyme families and metabolic pathways to assess regional differences in functional potential; and (3) reconstruct species-level genomes (SGBs) to identify specific microbial lineages and genomic features underlying community-level differences.

## 2. Materials and Methods

### 2.1. Animals and Sample Collection

Fresh feces were collected from wild adult red deer between 15 and 25 September 2025, in two geographical regions of China: Gaogesitai Hanwula Nature Reserve, Inner Mongolia Autonomous Region (NMG group; *n* = 16; 119°03′ E, 44°41′ N; elevation approximately 800–1200 m a.s.l.), and Sunan Yugur Autonomous County, Gansu Province (GS group; *n* = 16; 99°36′ E, 38°48′ N; elevation approximately 2600–3000 m a.s.l.). The NMG sampling site is located within mixed coniferous–broadleaf forests and forest–steppe transition zones, characterized by a temperate semi-arid continental monsoon climate. In this habitat, the naturally available dietary resources for red deer are dominated by woody browse, shrub shoots, and fibrous grasses. In contrast, the GS sampling site is situated in alpine meadows and montane forest edges with a cold alpine semi-arid climate, providing a diet richer in herbaceous plants, sedges, and alpine forbs. A non-invasive sampling strategy combined with direct visual observation and tracking of individual red deer was employed [29]. Fresh feces were collected immediately after natural defecation was observed. Sample freshness was determined based on the following criteria: a moist surface, smooth appearance, well-formed rounded pellets, and minimal adhesion between adjacent pellets [30,31]. However, due to the limitations of the non-invasive sampling strategy in the wild, the exact age, sex, and genetic identity of the individuals could not be determined. To reduce the possibility of repeated sampling from the same individual, sampling was conducted across four to five distinct herds within each region. Moreover, only one sample was collected per defecation event, and individual samples were distinguished based on direct field observations and a minimum spatial separation of 50 m between sampling points. During collection, disposable polyethylene gloves were worn and replaced between samples to prevent cross-contamination. The outer layer of each fecal pellet was removed using a sterile surgical scalpel, and only the inner portion was retained for downstream analyses to minimize environmental contamination. Feces were immediately snap-frozen in liquid nitrogen for transport to the laboratory and subsequently stored at −80 °C. This study was approved by the Animal Welfare and Ethics Committee of Jilin Agricultural University (Approval No. 20230421001).

### 2.2. Fecal Microbial DNA Extraction

Total microbial genomic DNA was extracted from 200 mg of each fecal sample using the TIANamp Stool DNA Kit (DP328; Tiangen Biotech Co., Ltd., Beijing, China) following the manufacturer’s protocol. DNA concentration and purity were assessed using a BioTek Epoch 2 microplate spectrophotometer (BioTek Instruments, Inc., Winooski, VT, USA). DNA integrity was further examined by 1% agarose gel electrophoresis. The qualified DNA extracts were aliquoted and stored at −80 °C until further analysis.

### 2.3. 16S rRNA Gene Sequencing and Bioinformatic Analysis

The V3–V4 regions of the microbial 16S rRNA gene were amplified using the primer pair 341F (5′-CCTAYGGGRBGCASCAG-3′) and 806R (5′-GGACTACNNGGGTATCTAAT-3′) [32]. The resulting PCR amplicons were purified using AMPure XP magnetic beads (A63881; Beckman Coulter, Brea, CA, USA) and quantified with a Qubit 3.0 Fluorometer (Thermo Fisher Scientific, Waltham, MA, USA). Sequencing libraries were constructed using the NEBNext^®^ Ultra™ II DNA Library Preparation Kit (New England Biolabs, Ipswich, MA, USA). Library concentration was assessed using a Qubit^®^ 2.0 Fluorometer (Thermo Fisher Scientific, Waltham, MA, USA). Qualified libraries were subjected to paired-end sequencing (2 × 250 bp) on an Illumina NovaSeq 6000 (Illumina, San Diego, CA, USA).

Raw reads were processed using fastp (v0.23.1) for adapter removal and quality filtering [33]. The clean paired-end reads were then imported into QIIME 2 and processed with the DADA2 plugin, including quality filtering, denoising, paired-end merging, and chimera removal, to generate amplicon sequence variants (ASVs) [34]. Taxonomic classification of the representative sequence of each ASV was performed against the SILVA SSU rRNA database (release 138.1) [35]. Subsequent microbial community analyses were conducted using microeco (v1.15.0) [36]. Principal coordinate analysis (PCoA) was performed based on weighted UniFrac and Bray–Curtis dissimilarity matrices to evaluate differences in microbial community, and the statistical significance was determined by permutational multivariate analysis of variance (PERMANOVA) with 999 permutations. Spearman’s rank correlation coefficients between microbial genera were calculated using Hmisc (v5.2-6) [37]. Significant correlations were identified using the criteria of adjusted *p* ≤ 0.05 and |rho| > 0.5, and the resulting microbial correlation networks were visualized using Gephi (v0.10.1) [38].

### 2.4. Metagenomic Sequencing and Bioinformatic Analysis

For each sample, 1 μg of genomic DNA was used to construct sequencing libraries with an average insert size of approximately 350 bp using the NEBNext^®^ Ultra™ II DNA Library Preparation Kit (New England Biolabs, MA, USA). The resulting libraries were sequenced on an Illumina NovaSeq 6000 platform (Illumina, San Diego, CA, USA) to generate 150 bp paired-end reads. Raw reads were subjected to quality filtering using fastp (v0.23.1) [33]. Host-derived sequences were removed by mapping the quality-filtered reads against the red deer reference genome [39] using Bowtie (v2.3.5.1) [40]. The resulting non-host reads were then de novo assembled for each sample using MEGAHIT (v1.2.9) [41]. Open reading frames (ORFs) were predicted using Prodigal (v2.6.3), and ORFs shorter than 100 bp were discarded [42]. The predicted ORFs were clustered using CD-HIT (v4.8.1) [43] at a nucleotide sequence identity of 95% and a minimum sequence coverage of 90% to construct a non-redundant gene catalog. Quality-filtered non-host reads were mapped against the non-redundant gene catalog using BWA-MEM (v0.7.17) [44]. The resulting alignments were recorded in SAM format, and only uniquely mapped reads were retained for gene abundance quantification. Gene abundance was normalized as transcripts per million (TPM). Functional annotation of the non-redundant gene catalog was performed using DIAMOND (v2.1.6) [45] against the Kyoto Encyclopedia of Genes and Genomes (KEGG) database (v108.0) [46]. CAZyme families were annotated using dbCAN3 [47]. For downstream analyses, only KEGG Orthology (KO) terms with a prevalence > 10% across all samples (a total of 4594 KOs) were retained. Finally, KO enrichment analysis was performed using clusterProfiler 4.0 [48].

Genome reconstruction was performed on the contigs generated from the individual assembly of each fecal sample using MetaBAT2 and MaxBin2 integrated within the MetaWRAP (v1.3.2) [49]. Quality-controlled clean reads from each sample were mapped to the assembled contigs using BWA-MEM, and the resulting alignments were sorted into BAM files using SAMtools (v1.5) [50]. Genome quality was assessed using CheckM (v1.0.7) [51] and classified according to the MIMAG criteria [52]. The rRNA and tRNA genes were identified using Barrnap (v1.10.5) [53] and ARAGORN (v1.2.41) [54], respectively. MAGs with completeness ≥50% and contamination ≤10% were retained for subsequent analyses. The resulting MAGs were dereplicated using dRep (v3.9) [55] at a 95% average nucleotide identity (ANI) threshold to obtain SGBs. Potential genome chimerism of the SGBs was evaluated using GUNC (v1.1.1) [56] against the proGenomes 2.1 database [57]. The relative abundance of each SGB across samples was quantified using the Quant_bins module in MetaWRAP. Only SGBs with a mean relative abundance > 0.1% across all samples were retained for the comparison. Taxonomic classification of the SGBs was performed using GTDB-Tk (v2.3.1) [58]. A phylogenetic tree of the SGBs was constructed using FastTree (v2.1.11) [59] and visualized using tvBOT (v2.6.1) [60]. Statistical significance of differences in microbial community structure between groups was evaluated by PERMANOVA with 999 permutations. Canonical correspondence analysis (CCA) was performed using vegan (v2.6–6) [61]. The significance of the overall CCA model and individual canonical axes was assessed using 999 permutations.

### 2.5. Statistical Analysis

All statistical analyses were performed in R (v4.3.1). Differences in alpha-diversity indices and the relative abundances of taxa, CAZyme families, KO terms, KEGG pathways, and SGBs between the NMG and GS groups were assessed using the Wilcoxon rank-sum test. *p* values were adjusted using the Benjamini–Hochberg procedure to control the false discovery rate. An adjusted *p* value < 0.05 was considered statistically significant. Homogeneity of multivariate dispersions was evaluated using the betadisper function from the vegan package followed by permutation-based tests with 999 permutations for each distance matrix. All data are presented as the mean ± standard error of the mean.

## 3. Results

### 3.1. Comparison of Fecal Microbial Composition Between the GS and NMG Groups

**Figure 1:**
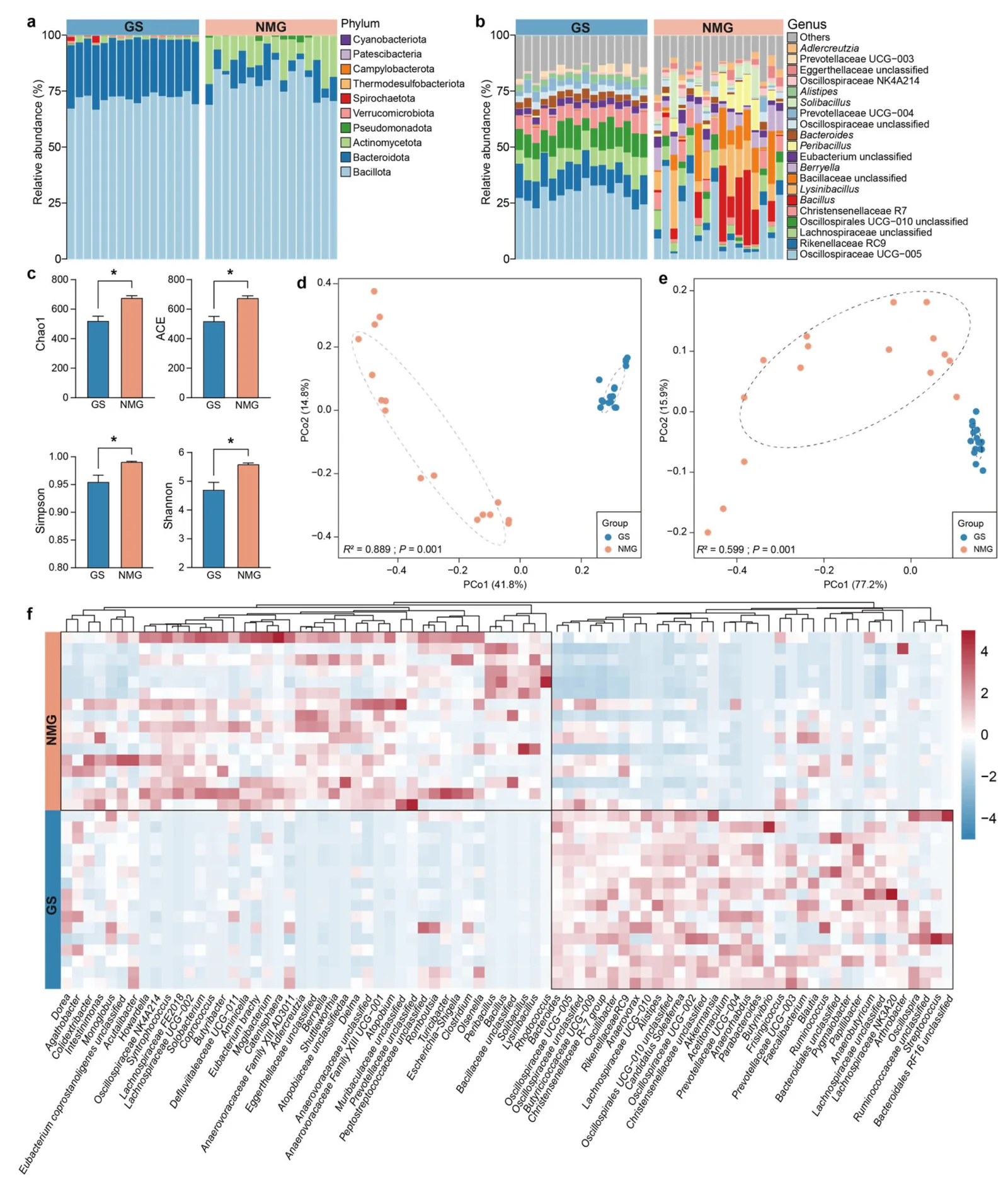
Fecal microbial community and differences between the GS and NMG groups. Relative abundance of fecal microbial communities at the phylum (**a**) and the genus (**b**) levels in the GS and NMG groups. Square colors indicate the taxonomic classification. (**c**) Comparison of alpha-diversity indices of fecal microbial communities between the GS and NMG groups. PCoA based on Bray–Curtis dissimilarity (**d**) and weighted UniFrac distances (**e**) showing differences in fecal microbial communities between the GS and NMG groups. Dashed ellipses represent the 95% confidence intervals of each group. (**f**) Heatmap showing the significantly different genera between the GS and NMG groups. * indicates adjusted *p* < 0.05.

A total of 32 fecal samples (16 per group) were subjected to 16S rRNA gene sequencing, and 3,396,981 high-quality reads were obtained, with an average of 106,155 ± 1010 reads per sample. In total, 1701 ASVs were identified and taxonomically assigned to 10 phyla, 14 classes, 34 orders, 57 families, and 149 genera. At the phylum level, the predominant phyla in both groups were Bacillota (NMG, 78.88 ± 6.43%; GS, 71.71 ± 2.34%), Bacteroidota (NMG, 8.09 ± 5.41%; GS, 26.07 ± 1.76%), and Actinomycetota (NMG, 12.26 ± 5.95%; GS, 1.18 ± 0.86%; Figure 1a). At the genus level, Oscillospiraceae UCG-005 was the most abundant taxon in the NMG group (16.28 ± 13.73%), followed by *Bacillus* (9.32 ± 11.93%), *Lysinibacillus* (8.43 ± 8.28%), and unclassified Bacillaceae (7.65 ± 8.74%). In the GS group, Oscillospiraceae UCG-005 was also the most abundant taxon (29.13 ± 4.12%), followed by Rikenellaceae RC9 (11.19 ± 2.14%), unclassified Oscillospirales UCG-010 (10.47 ± 2.43%), and unclassified Lachnospiraceae (8.18 ± 2.29%; Figure 1b).

Comparison revealed that the Chao1, ACE, Shannon, and Simpson indices of the fecal microbial community were significantly higher in the NMG group than in the GS group (adjusted *p* < 0.05; Figure 1c). PCoA based on Bray–Curtis dissimilarity (Figure 1d) and weighted UniFrac distances (Figure 1e) revealed clear separation of the fecal microbial community between the two groups, while a high inter-individual variability was observed in the NMG group. PERMANOVA further confirmed significant between-group differences based on both Bray–Curtis dissimilarity (*R*^2^ = 0.889, adjusted *p* = 0.001) and weighted UniFrac distances (*R*^2^ = 0.599, adjusted *p* = 0.001). Significant heterogeneity within groups was confirmed for both Bray–Curtis (*F* = 6.599, adjusted *p* = 0.024) and weighted UniFrac (*F* = 48.561, adjusted *p* = 0.001) via betadisper analysis. Co-occurrence network analysis indicated that the fecal microbial network of the NMG group exhibited greater structural complexity than that of the GS group (Figure S1).

Furthermore, the comparison identified 80 genera with significantly different relative abundances between the NMG and GS groups (adjusted *p* < 0.05; Figure 1f). The relative abundances of *Rhodococcus*, *Lysinibacillus*, *Bacillus*, *Olsenella*, *Clostridium*, *Escherichia*, Anaerovoracaceae UCG-001, *Eubacterium*, *Aminicella*, *Butyrivibrio*, *Coprococcus*, Lachnospiraceae UCG-002, and Oscillospiraceae NK4A214 were higher in the NMG group than in the GS group (adjusted *p* < 0.05). In contrast, the GS group exhibited higher relative abundances of *Streptococcus*, *Oscillospira*, Lachnospiraceae NK3A20, *Ruminococcus*, Prevotellaceae UCG-003, *Faecalibacterium*, *Parabacteroides*, Prevotellaceae UCG-004, *Akkermansia*, Oscillospiraceae UCG-002, *Alistipes*, Lachnospiraceae UCG-010, Rikenellaceae RC9, *Oscillibacter*, Christensenellaceae R7, Oscillospiraceae UCG-005, and *Bacteroides* compared with the NMG group (adjusted *p* < 0.05).

### 3.2. Metagenomic Sequencing Summary and Annotation

Metagenomic sequencing of the red deer feces generated 269 Gb of sequencing reads, with an average of 8.4 Gb per sample. De novo assembly yielded 20,130,069 contigs, from which Prodigal predicted 33,581,832 ORFs. After clustering at a 95% nucleotide sequence identity, a non-redundant fecal microbial gene catalog comprising 6,181,482 genes was obtained. A total of 6, 46, and 462 metabolic pathways were identified at KEGG level 1, level 2, and level 3, respectively. Meanwhile, 325 CAZyme families were detected in the fecal microbiome (Figure S2a), spanning six enzyme classes: glycoside hydrolases (GHs, *n* = 144, 44.3%), glycosyltransferases (GTs, *n* = 67, 20.6%), carbohydrate-binding modules (CBMs, *n* = 61, 18.8%), polysaccharide lyases (PLs, *n* = 27, 8.3%), carbohydrate esterases (CEs, *n* = 17, 5.2%), and auxiliary activities (AAs, *n* = 9, 2.8%).

### 3.3. Comparison of Functional Potential in Feces Between the GS and NMG Groups

**Figure 2:**
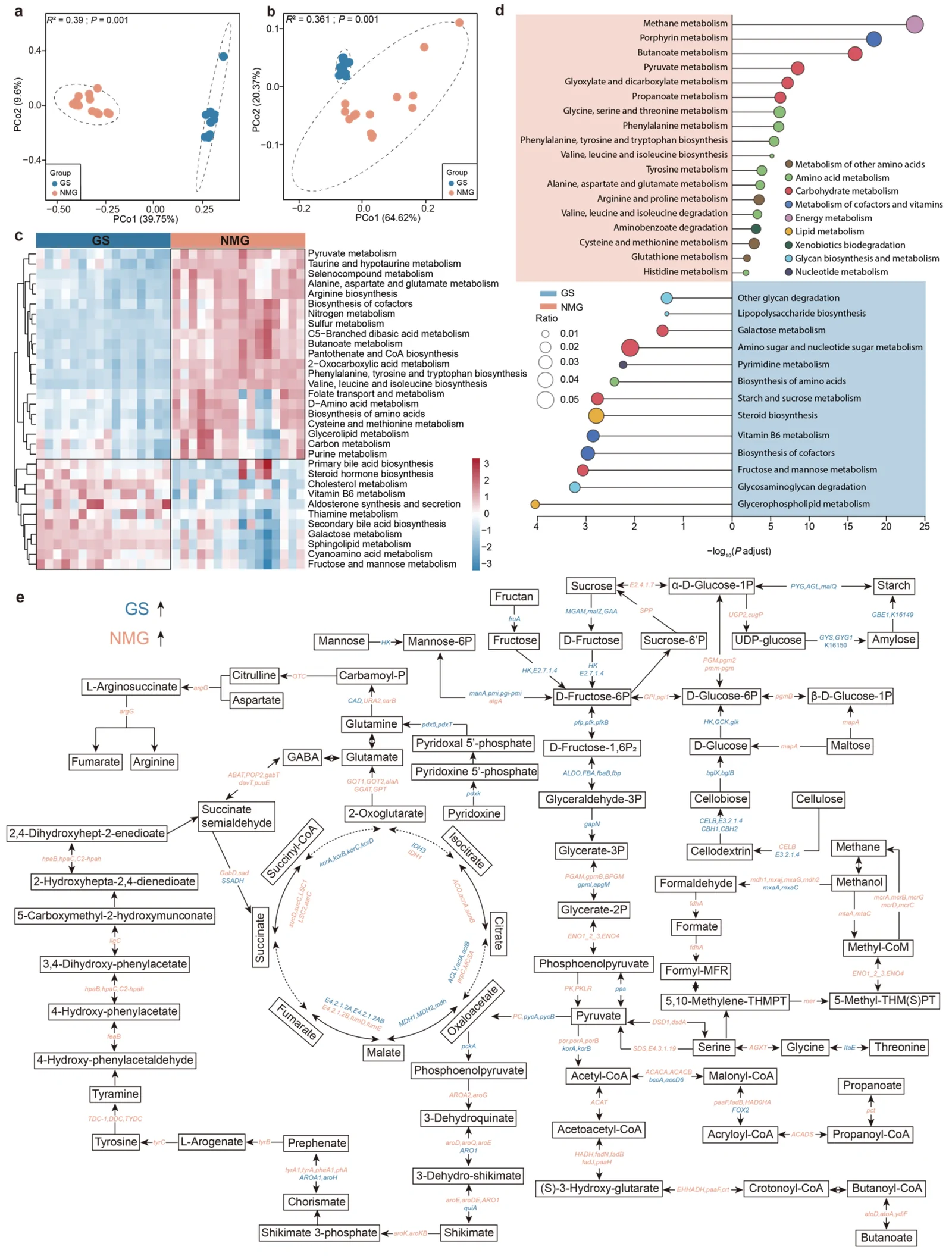
A comparison of the functional potential of the fecal microbiome between the GS and NMG groups. PCoA showing the CAZyme profiles (**a**) and KOs (**b**) based on the Bray–Curtis dissimilarity between the GS and NMG groups. Dashed ellipses represent the 95% confidence intervals of each group. (**c**) The significantly different metabolic pathways at KEGG level 3 between the GS and NMG groups. (**d**) The significantly enriched metabolic pathways in the fecal microbiome of the GS and NMG groups. Circle size represents the gene ratio, circle color represents the metabolic category at KEGG level2, and bar length represents the -log_10_ (adjusted *p* value). (**e**) The reconstructed metabolic pathways based on significantly different KOs in the fecal microbiome of the GS and NMG groups. Orange and blue indicate the significantly increased KOs in the NMG and GS groups, respectively.

PCoA revealed significant differences between the GS and NMG groups in both the CAZyme (*R*^2^ = 0.390, adjusted *p* = 0.001; Figure 2a) and KO profiles (*R*^2^ = 0.361, adjusted *p* = 0.001; Figure 2b). Tests for homogeneity showed uniform within-group variances for the CAZyme profile (*F* = 1.851, adjusted *p* = 0.174), whereas significant within-group heterogeneity was observed for the KO profile (*F* = 10.071, adjusted *p* = 0.001). Comparison revealed that eight metabolic pathways differed significantly in relative abundance between the two groups at KEGG level 2 (Figure S2b). At KEGG level 3, 32 pathways were significantly different between the two groups (Figure 2c). Pathways enriched in the NMG group included pyruvate metabolism; butanoate metabolism; taurine and hypotaurine metabolism; biosynthesis of cofactors; biosynthesis of amino acids (arginine, phenylalanine, tyrosine, tryptophan, valine, leucine, and isoleucine); folate biosynthesis; alanine, aspartate, and glutamate metabolism; cysteine and methionine metabolism; glycerolipid metabolism; and purine metabolism (adjusted *p* < 0.05). In contrast, the GS group showed significantly higher abundances of fructose and mannose metabolism, galactose metabolism, primary and secondary bile acid biosynthesis, cyanoamino acid metabolism, thiamine metabolism, and vitamin B_6_ metabolism (adjusted *p* < 0.05).

Further comparison showed that a total of 2582 KOs were different between the two groups (adjusted *p* < 0.05; Table S1). KO enrichment analysis (Figure 2d) revealed that the increased KOs enriched in the NMG group were primarily involved in butanoate, pyruvate, and propanoate metabolism; glycine, serine, and threonine metabolism; methane metabolism; biosynthesis of amino acids (phenylalanine, tyrosine, tryptophan, valine, leucine, and isoleucine), amino acid metabolism (tyrosine, alanine, aspartate, glutamate, arginine, proline, cysteine, methionine, glutathione, and histidine); and valine, leucine, and isoleucine degradation. In contrast, KOs enriched in the GS group were mainly associated with starch and sucrose metabolism, galactose metabolism, lipopolysaccharide biosynthesis, amino sugar and nucleotide sugar metabolism, pyrimidine metabolism, steroid biosynthesis, vitamin B_6_ metabolism, fructose and mannose metabolism, glycosaminoglycan degradation, glycerophospholipid metabolism, amino acid biosynthesis, and cofactor biosynthesis.

We then reconstructed the metabolic pathways of the fecal microbiome based on the differentially enriched KOs (Figure 2e). In the NMG group, enriched KOs were mapped to the biosynthesis of phenylalanine, tyrosine, and tryptophan (*tyrC*, *tyrB*); glycine, serine, and threonine metabolism (*AGXT*, *DSD1*); arginine and proline metabolism (*argG*); alanine, aspartate, and glutamate metabolism (*GOT1*, *GOT2*, *GPT*); butanoate metabolism (*atoD*, *atoA*, *ydiF*); and propanoate metabolism (*pct*, *ACADS*, *paaF*, *fadB*). In the GS group, enriched KOs were predominantly mapped to starch and sucrose metabolism (*PYG*, *GBE1*, *malQ*), fructose and mannose metabolism (*truA*, *HK*), other glycan degradation (*CBH1*, *CBH2*), and vitamin B6 metabolism (*pdxK*, *pdx5*, *pdxT*).

### 3.4. Comparison of CAZymes in Feces Between the GS and NMG Groups

**Figure 3:**
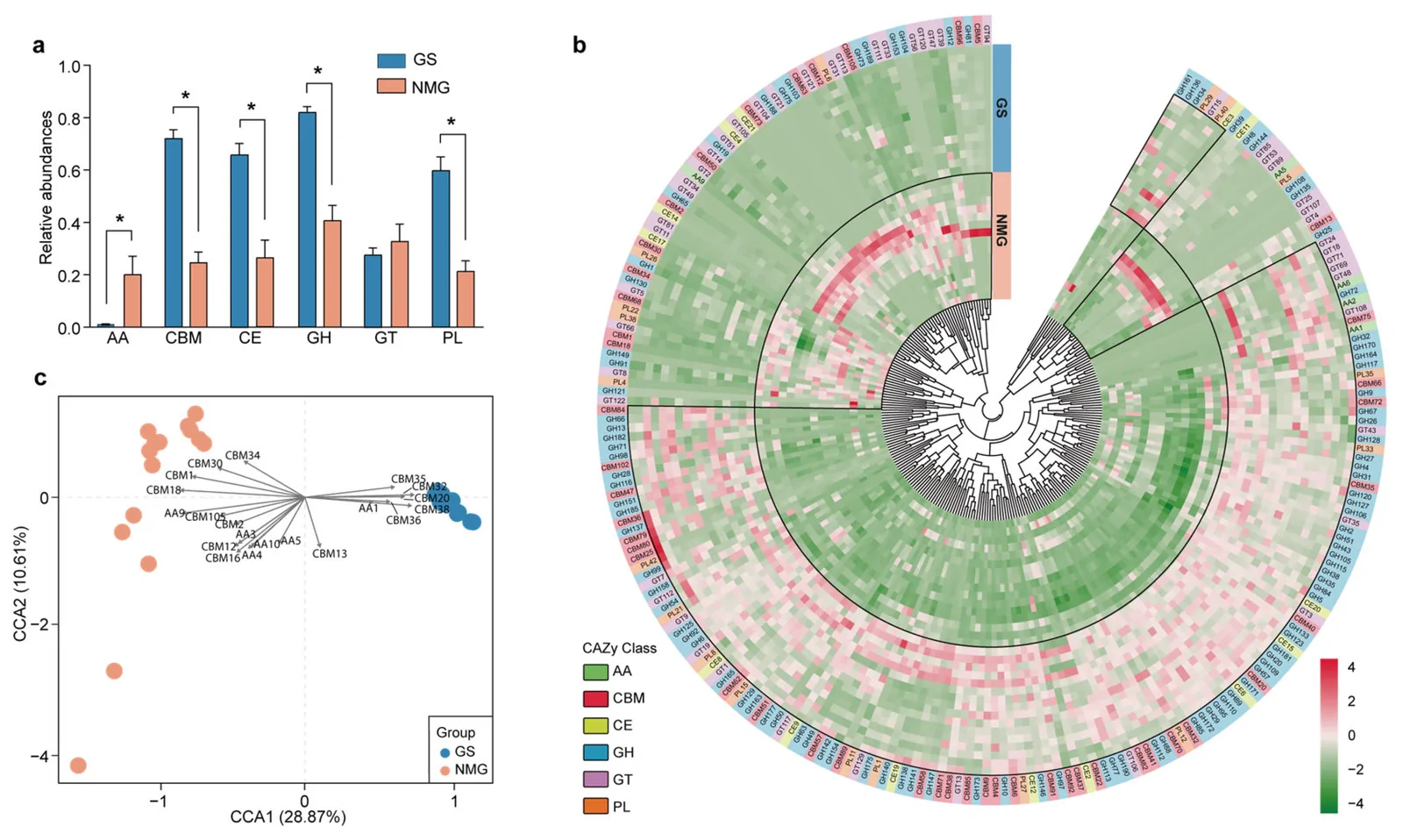
A comparison of CAZyme families in the fecal microbiome between the GS and NMG groups. (**a**) A comparison of the main category of CAZyme families between the GS and NMG groups. (**b**) A heatmap showing the different CAZyme families between the GS and NMG groups. The colors of the outer ring indicate the corresponding CAZyme family categories. (**c**) CCA showing the specific CAZyme in the fecal microbiome of the GS and NMG groups. * indicates adjusted *p* < 0.05.

Comparison of CAZyme family abundances between the two groups revealed that the relative abundances of genes encoding CBMs, CEs, GHs, and PLs were significantly higher in the GS group than in the NMG group, whereas the relative abundance of genes encoding AAs was significantly lower in the GS group (Figure 3a; adjusted *p* < 0.05).

Comparison revealed that the GS group was significantly enriched in gene families involved in the degradation and binding of multiple plant cell wall polysaccharides (adjusted *p* < 0.05), including cellulose (GH5, GH6, GH9, GH48, CBM4, CBM6, and CE4), hemicellulose (GH10, GH43, GH51, GH54, GH115, and GH120), pectin (GH28, GH105, PL1, PL27, PL40, and PL42), and starch (GH13, GH57, GH97, GH133, and CBM20). In contrast, the NMG group was significantly enriched in gene families associated with lignin modification (AA1 and AA2), additional cellulose-associated families (GH1, GH12, CBM1, CBM2, and CBM30), pectin-degrading families (PL4, PL5, PL6, and PL38), and chitin-associated families (GH19, CE14, CBM12, and CBM73). Furthermore, the NMG group showed significant enrichment in starch-binding modules (CBM34 and CBM68), enzymes related to trehalose metabolism (GH65), and glycosyltransferases involved in carbohydrate synthesis (GT5; adjusted *p* < 0.05; Figure 3b). CCA revealed that specific CAZy families exhibited characteristic associations with different groups. Among these, families such as CBM34, CBM30, CBM1, CBM18, CBM105, CBM2, AA9, AA3, AA4, AA5, and AA10 were primarily associated with the NMG group, while families including CBM35, CBM32, CBM20, CBM38, and CBM13 showed stronger correlations with the GS group (Figure 3c).

### 3.5. Comparison of SGBs in the NMG and GS Groups

**Figure 4:**
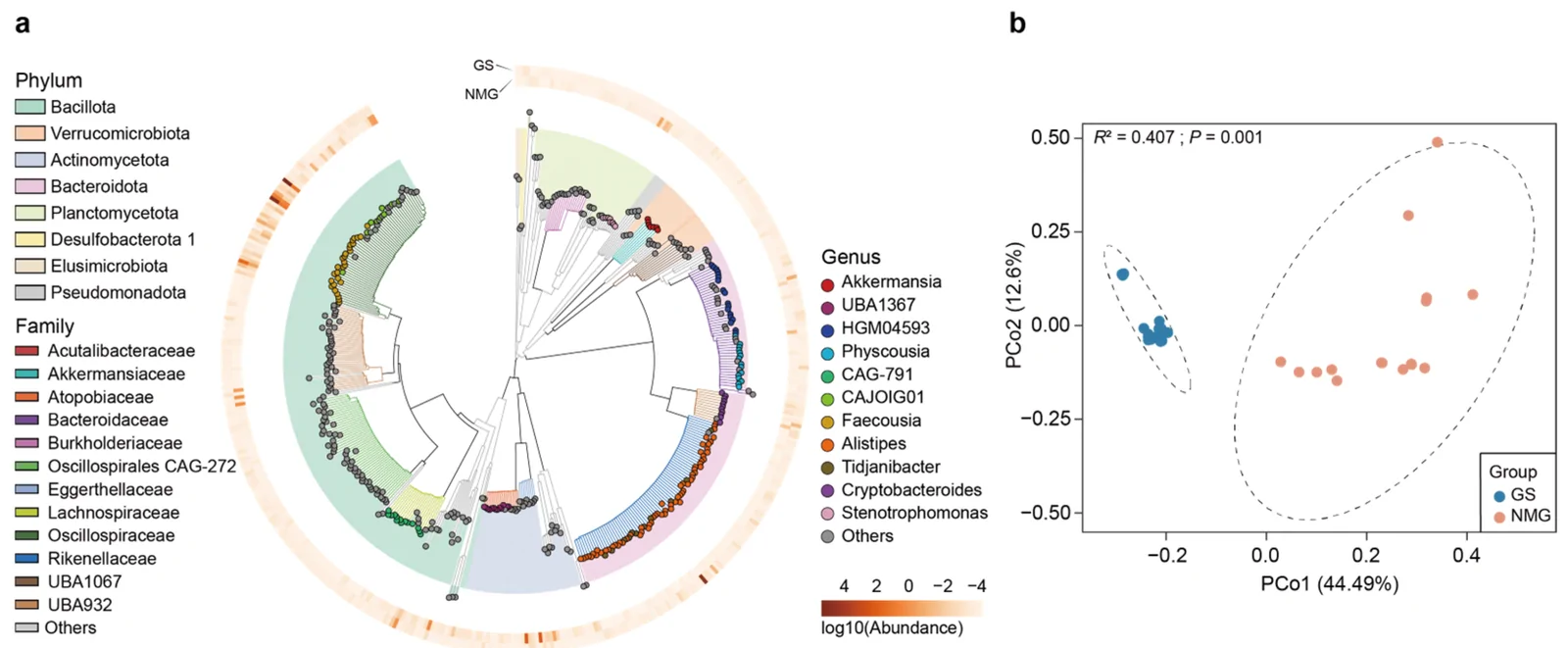
The assembled SGBs from the feces of red deer. (**a**) The phylogenetic tree of the 456 SGBs. The colors of the background and branches represent the taxonomic classifications at the phylum and family levels, respectively. The circle colors indicate genus-level classification. The two outer bars represent the relative abundance of each SGB in the NMG and GS groups, respectively. (**b**) PCoA of fecal SGB profiles between the NMG and GS groups based on the Bray–Curtis dissimilarity. Dashed ellipses represent the 95% confidence intervals of each group.

Using a single-sample assembly approach, a total of 2482 MAGs were reconstructed from the red deer feces. After quality filtering, 711 medium-quality and 95 high-quality MAGs were retained. Dereplication using dRep at a 95% ANI threshold yielded 456 SGBs, of which 430 (94.3%) passed the chimera assessment, while 26 (5.7%) were flagged as potentially chimeric (Table S2). Taxonomic classification with GTDB-Tk assigned the 456 SGBs to 8 phyla, 29 orders, 49 families, and 118 genera (Table S2). At the phylum level, 190 SGBs were affiliated with Bacillota, 143 with Bacteroidota, 47 with Pseudomonadota, 42 with Actinomycetota, and 25 with Verrucomicrobiota (Figure S2c). At the genus level, 456 SGBs were composed of *Alistipes* (*n* = 64), *Faecousia* (*n* = 28), Bacteroidaceae HGM04593 (*n* = 18), *Physcousia* (*n* = 15), and Lachnospiraceae CAG-791 (*n* = 14) (Figure 4a). PCoA revealed a significant difference in SGB composition between the GS and NMG groups based on Bray–Curtis dissimilarity (*R*^2^ = 0.407, adjusted *p* = 0.001; Figure 4b), with no significant differences in within-group dispersion (*F* = 0.002, adjusted *p* = 0.973).

**Figure 5:**
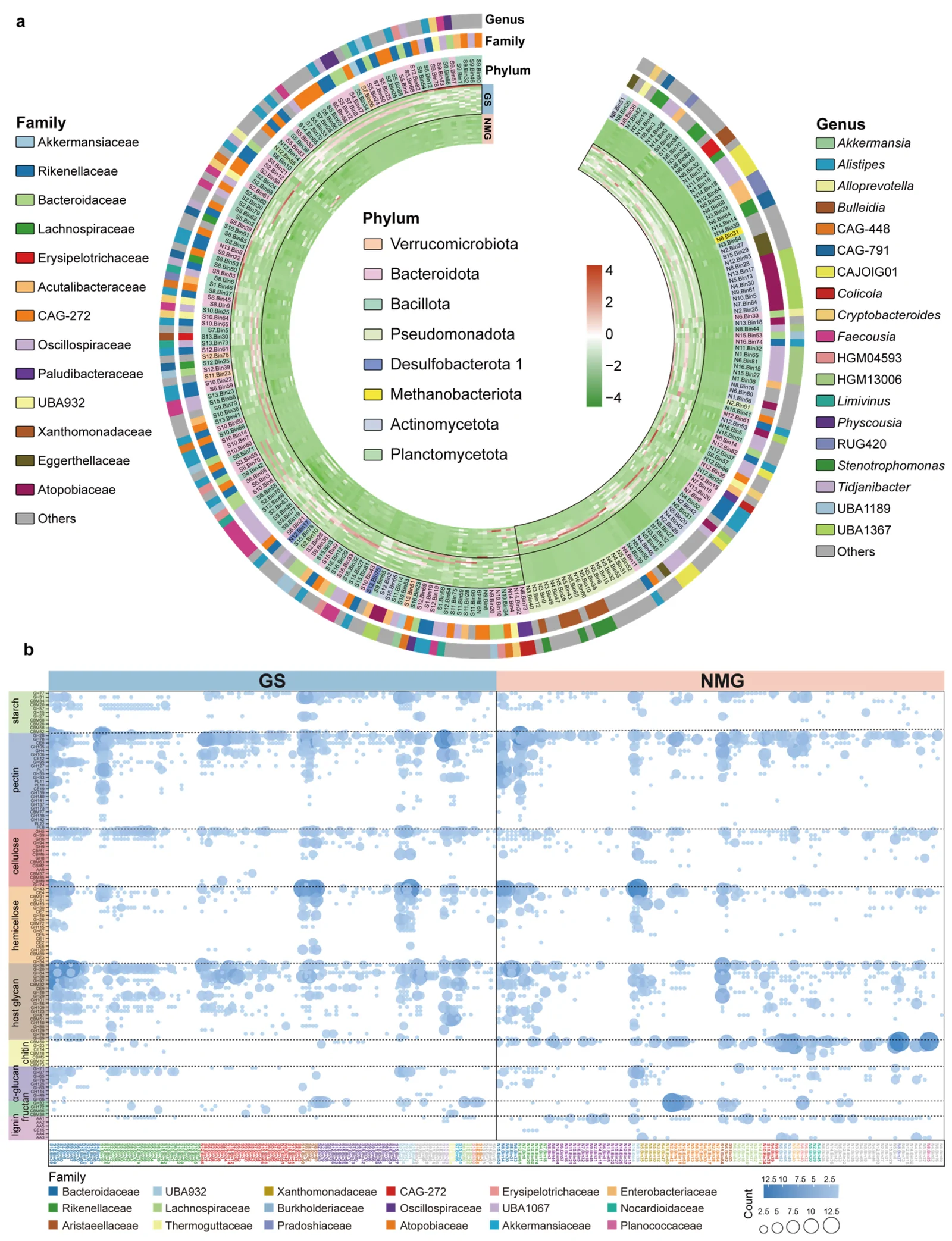
A comparison of fecal SGBs between the GS and NMG groups of red deer. (**a**) A heatmap of SGBs showing significant differences between the GS and NMG groups. The colors of the circles, from inner to outer, represent the taxonomic levels of phylum, family, and genus of the differentially abundant SGBs, respectively. (**b**) A comparison of CAZyme-encoding genes in the differentially abundant SGBs between the GS and NMG groups. Circle size and color represent the count of genes. These counts were used for descriptive visualization and were not interpreted as normalized estimates of functional capacity across genomes.

Comparative analysis identified 240 SGBs with significantly different abundances between the two groups (Figure 5a), of which 133 were enriched in the GS group and 107 in the NMG group (adjusted *p* < 0.05). Of these, 133 were enriched in the GS group (comprising 117 medium-quality and 16 high-quality SGBs), while 107 were enriched in the NMG group (comprising 93 medium-quality and 14 high-quality SGBs). The SGBs enriched in the GS group predominantly belonged to Rikenellaceae (*n* = 30), Oscillospirales CAG-272 (*n* = 30), Bacteroidaceae (*n* = 17), Akkermansiaceae (*n* = 2), and UBA1067 (*n* = 2), whereas those enriched in the NMG group were primarily affiliated with Atopobiaceae (*n* = 12), Lachnospiraceae (*n* = 7), Acutalibacteraceae (*n* = 6), Eggerthellaceae (*n* = 5), Anaerovoracaceae (*n* = 4), and Bifidobacteriaceae (*n* = 2). Among the GS-enriched SGBs, CAZyme genes associated with pectin degradation were frequently detected in SGBs affiliated with Rikenellaceae and Oscillospirales CAG-272, whereas CAZyme genes associated with starch and hemicellulose degradation were detected in SGBs affiliated with Oscillospiraceae and Bacteroidales UBA932. In contrast, several NMG-enriched SGBs affiliated with Burkholderiaceae, Planococcaceae, and Pradoshiaceae encoded CAZyme genes associated with lignin and chitin degradation (Figure 5b).

## 4. Discussion

In this study, 16S rRNA gene sequencing and metagenomic analysis revealed that the fecal microbiome of wild red deer was dominated by Bacillota (known as Firmicutes) and Bacteroidota (known as Bacteroidetes), which is consistent with previous reports on captive and free-ranging red deer [4,6,14,17,19,23,24,62,63]. Bacillota are primarily involved in the fermentation of plant-derived structural polysaccharides [64], whereas Bacteroidota harbor diverse glycoside hydrolases and polysaccharide utilization loci that enable the degradation of a wide range of complex plant-derived carbohydrates [65]. It has been revealed that wild red deer’s intake of plant fiber and carbohydrates was positively correlated with Firmicutes abundance in the feces [66], and the high abundance of Firmicutes in the gut was likely associated with adaptation to high fiber diets in roe deer (*Capreolus pygargus*) and moose (*Alces alces*) [67,68]. These results suggest a conserved microbial community in the gut of wild red deer, and that gut Firmicutes has a potential role in wild red deer adaptation. Despite the conserved dominant microbiota, we observed significant differences in microbial composition and genetic potential between the two groups. Luis et al. found that the free-living red deer had a more heterogeneous gut microbiome community than their captive counterparts [63]. Menke et al. revealed significant differences in the fecal microbiome between free-ranging red deer and those in winter enclosures in the Bavarian Forest National Park, with variations in Firmicutes abundance likely driven by silage consumption in captivity [24]. Similarly, comparisons between pre-released and wild red deer showed that increased plant fiber and carbohydrate intake significantly altered Firmicutes abundance [66]. Winter supplementary feeding has also been shown to enrich microbial pathways associated with butyrate and lipid metabolism [62]. These results indicate that dietary composition is a critical factor affecting the gut microbiome of wild deer. Classified as intermediate feeders, red deer consume a diverse array of plant species, ranging from graminoids, forbs, and fruits to the leaves, needles, and bark of woody plants [69]. The spatial locations influence variation in the vegetation available for feeding [70]. It has been observed that the difference in the gut microbiome of red deer at forest sites might partly be attributable to the higher variation in local food sources, both spatially and in terms of altitude [20,24]. These findings suggest that geographic differentiation may play a crucial role in shaping the fecal microbiome of wild red deer.

Importantly, the gut microbial ecosystem is highly dynamic and is also influenced by genetic background, environmental conditions, habitat type, sex, and age [6]. The fecal microbiome of hybrids between *Cervus elaphus songaricus* and *C. e. yarkandensis* closely resembled that of their paternal line, featuring increased abundances of Prevotellaceae and *Succinivibrio*, along with enriched pathways for steroid biosynthesis and vitamin metabolism (tryptophan, riboflavin, and vitamin B_6_) [71]. Guo et al. demonstrated that transitioning to wild environments significantly restructured the fecal microbial composition of released red deer over time [23], and reported significantly increased abundances of *Clostridium* in late winter compared to early winter [19]. In wild red deer, fecal microbial diversity was found to be higher in females than in males [72]. The juvenile forest musk deer harbored higher abundances of Clostridiales, whereas adults showed higher proportions of Lachnospiraceae [73]. Thus, the fecal microbiome of red deer represents a dynamic, adaptive ecosystem shaped by confounding factors including diet, habitat, environmental conditions, genetics, age, and sex.

Our results revealed higher abundances of *Clostridium*, *Eubacterium*, and *Bacillus*, which are known to degrade complex carbohydrates in the gut [74], in the feces of NMG red deer. This was accompanied by an enrichment of AA family enzymes and SGBs harboring CAZyme genes involved in lignin modification and chitin degradation. *Clostridium* is reported to degrade complex polysaccharides and structurally simple carbohydrates [75]. A higher proportion of *Bacillus* has been found in the feces of wild red deer compared to captive populations [14]. Notably, AA family enzymes oxidize lignin-related aromatics and phenolics [76]. Because lignin forms a recalcitrant matrix with cellulose and hemicellulose that impedes plant cell wall digestion [77], the enrichment of AAs is highly relevant to their diet. Red deer in Inner Mongolia typically inhabit temperate forest and forest–steppe transition zones, where diets include woody twigs and shrub shoots [12]. These results suggest that the fecal microbiome of red deer in NMG likely possesses an increased genetic potential for lignin modification and chitin degradation. In contrast, the feces of GS red deer exhibited an increase in *Ruminococcus*, *Oscillospira*, and *Faecalibacterium*, alongside an increase in GH and PL families involved in the degradation of cellulose, hemicellulose, and pectin. *Ruminococcus* is a common genus in the feces of wild sika deer [78] and captive red deer in South Korea [79] and Germany [63]; as a primary cellulose-degrading bacterium within the phylum Bacillota, it can degrade plant cellulose to produce acetate [80,81]. Most *F. prausnitzii* strains have the ability to utilize pectin for growth and compete for pectin as a substrate in the presence of other known pectin-utilizing species (e.g., *Eubacterium eligens*), indicating this genus contributes to pectin fermentation in the human colon [82]. The available plant resources in the alpine and montane ecosystems of Gansu are dominated by grasses and sedges, which are rich in both structural and non-structural carbohydrates [15,16]. These findings indicate that the fecal microbiome of red deer in GS is likely associated with the degradation of cellulose, hemicellulose, and pectin.

We also revealed pathway enrichment for the biosynthesis of branched-chain (Val, Leu, Ile) and aromatic (Phe, Tyr, Trp) amino acids in the NMG group, suggesting greater functional potential for *de novo* amino acid biosynthesis and nitrogen assimilation. This functional configuration may support metabolic potential under dietary conditions characterized by high carbon-to-nitrogen ratios and limited available nitrogen [83]. In addition, the fecal microbiome in the GS group showed enrichment of secondary bile acid metabolism and vitamin B_6_ metabolism. Microbial bile acid biotransformation generates a diverse array of secondary bile acids that not only affect lipid digestion but also act as signaling molecules regulating host glucose and lipid metabolism and energy homeostasis [84,85]. Pyridoxal 5′-phosphate, the active form of vitamin B_6_, serves as an essential cofactor in amino acid, carbohydrate, and redox metabolism [86]. These functional potentials likely contribute to adaptation of red deer inhabiting the cold, hypoxic environments of high-altitude regions.

It is worth noting that our study has several limitations. First, we cannot exclude the confounding effects of variations in plant composition and nutritional components between the two geographic regions. Therefore, future studies should employ dietary metabarcoding to profile food composition and elucidate its correlation with functional shifts in the gut microbiome. Second, fecal microbiome primarily represents the hindgut microbiome and is susceptible to various confounding factors, including season, age, sex, and genetic background. Furthermore, while metagenomics provides insights into the functional potential of the microbiome, it does not reflect actual metabolic activity. To address this, future research should integrate metatranscriptomics with targeted and untargeted metabolomics. Utilizing larger sample cohorts with controlled genetic backgrounds, ages, and sexes across different periods will help characterize the metabolic activities of the fecal microbiome and clarify its role in host adaptation. Finally, comprehensive host phenotypic assessments including body condition and blood parameters are needed to determine whether the observed fecal microbiome divergence represents potential adaptive microbial responses and whether these differences confer physiological or fitness benefits to wild red deer.

## 5. Conclusions

This study revealed differences in the community structure and functional potential of the fecal microbiome of wild red deer inhabiting Inner Mongolia and Gansu Province. The fecal microbiome of Gansu red deer was characterized by enrichment of functional potential related to the utilization of cellulose, hemicellulose, and pectin, together with pathways for secondary bile acid and vitamin B_6_ metabolism. In contrast, the fecal microbiome of Inner Mongolia red deer exhibited greater genetic potential associated with the utilization of lignified plant materials and amino acid biosynthesis. Together, these results demonstrate geographic differentiation of the fecal microbiome between the two wild red deer populations, suggesting potential adaptive microbial responses.

## Data Availability

The original data presented in the study are openly available in Genome Sequence Archive (GSA, https://ngdc.cncb.ac.cn/gsa, accessed on 20 August 2026) database project CRA049104 (16S rRNA gene sequences) and CRA049080 (shot-gun sequences).

## Supplementary Materials

The following supporting information can be downloaded at https://www.mdpi.com/article/10.3390/microorganisms14102404/s1. Figure S1: Co-occurrence network graphs and network topological parameters of fecal microbiota of red deer in the GS and NMG groups; Figure S2: Functional profiles and carbohydrate-active enzyme family composition of fecal microbial communities in the GS and NMG groups of red deer. (**a**) Composition of carbohydrate-active enzyme (CAZyme) families in the fecal microbiome of red deer. (**b**) KEGG level-2 metabolic pathways showing significant differences between the GS and NMG groups. (**c**) Phylum-level taxonomic composition of SGBs; Table S1: The different KOs between the GS and NMG groups; Table S2: The genome quality, taxonomic classification, and abundance of SGBs between the GS and NMG groups.

## Abbreviations

The following abbreviations are used in this manuscript.

CAZymes	Carbohydrate-active enzymes
MAG	Metagenome-assembled genome
SGB	Species-level genome
PERMANOVA	Permutational multivariate analysis of variance
PCoA	Principal coordinate analysis
ASV	Amplicon sequence variant
ORF	Open reading frame
TPM	Transcripts per kilobase million
KEGG	Kyoto Encyclopedia of Genes and Genomes
KO	KEGG Orthology
ANI	Average nucleotide identity
CCA	Canonical correspondence analysis
GH	Glycoside hydrolase
GT	Glycosyltransferase
CBM	Carbohydrate-binding module
PL	Polysaccharide lyase
CE	Carbohydrate esterase
AA	Auxiliary activity
