# Supplementary material for: Comparison of Fecal Microbiome of Wild Red Deer in Different Regions Reveals Geographic Differentiation
Source: Microorganisms. 2026 Oct 20;14(10):2404. doi: 10.3390/microorganisms14102404 (PMC13707525; doi:10.3390/microorganisms14102404)
Supplement: Supplementary file 1 [file microorganisms-14-02404-s001.zip › Supplementary Figures.pdf]

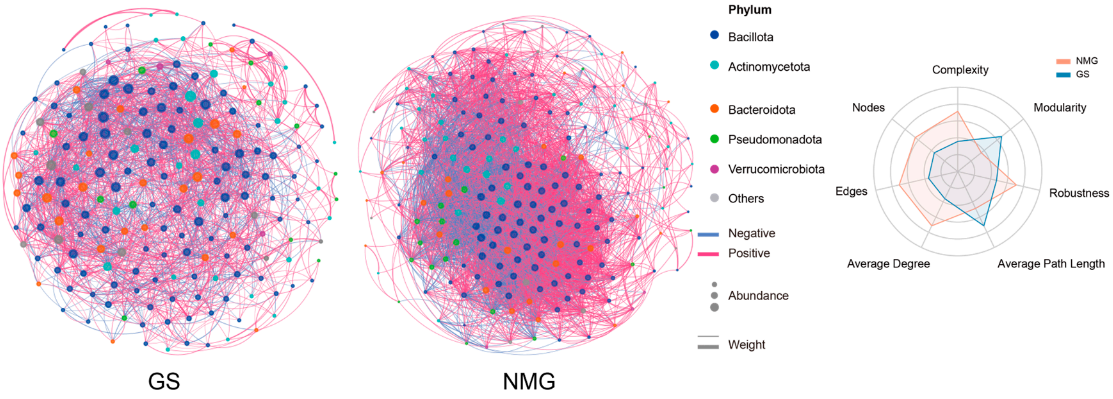

Figure S1. Co-occurrence network graphs and network topological parameters of fecal microbiota of red deer in the GS and NMG groups.

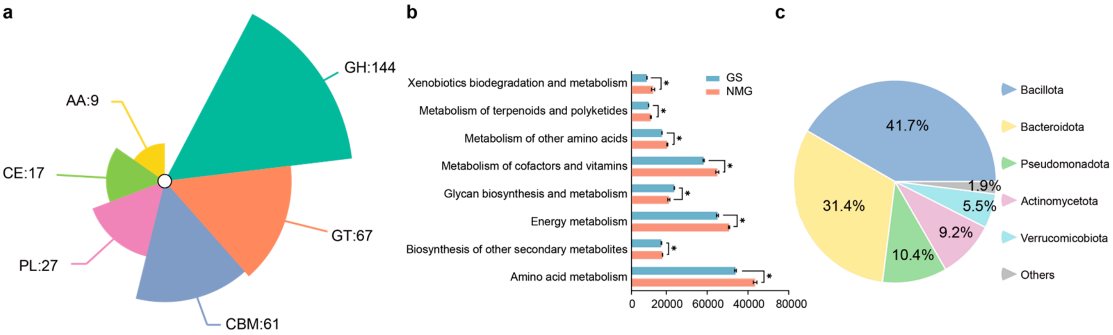

Figure S2. Functional profiles and carbohydrate-active enzyme family composition of fecal microbial communities in the GS and NMG groups of red deer. (**a**) Composition of carbohydrate-active enzyme (CAZyme) families in the fecal microbiome of red deer. (**b**) KEGG level-2 metabolic pathways showing significant differences between the GS and NMG groups. (**c**) Phylum-level taxonomic composition of SGBs.
